# Supplementary material for: Attachable Inertial Device with Machine Learning toward Head Posture Monitoring in Attention Assessment
Source: Micromachines (Basel). 2022 Dec 14;13(12):2212. doi: 10.3390/mi13122212 (PMC9788255; doi:10.3390/mi13122212)
Supplement: Supplementary file 1 [file micromachines-13-02212-s001.zip › micromachines-2066834-supplementary.pdf]

Attachable Inertial Device with Machine Learning toward Head-Posture Monitoring in Attention Assessment

Ying Peng, Chao He, Hongcheng Xu

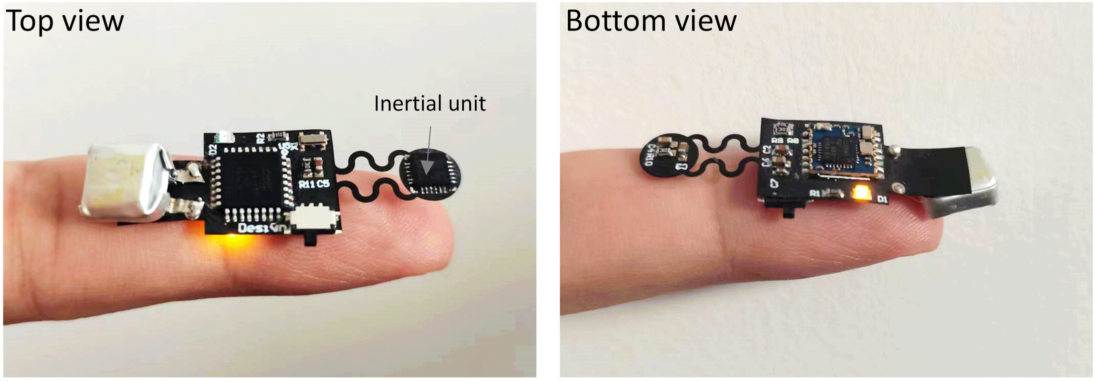

Figure S1. Optical images of the bare circuit of the attachable device on the finger, top view (left) and bottom view (right).

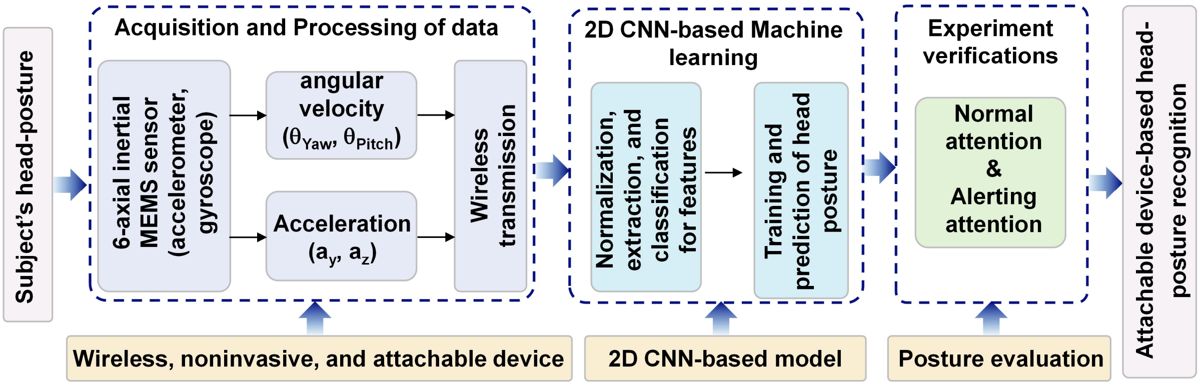

Figure S2. Flow block of the complete system that shows the attention evaluation from data acquisition and processing to posture training and prediction, finally to real-posture verifications.

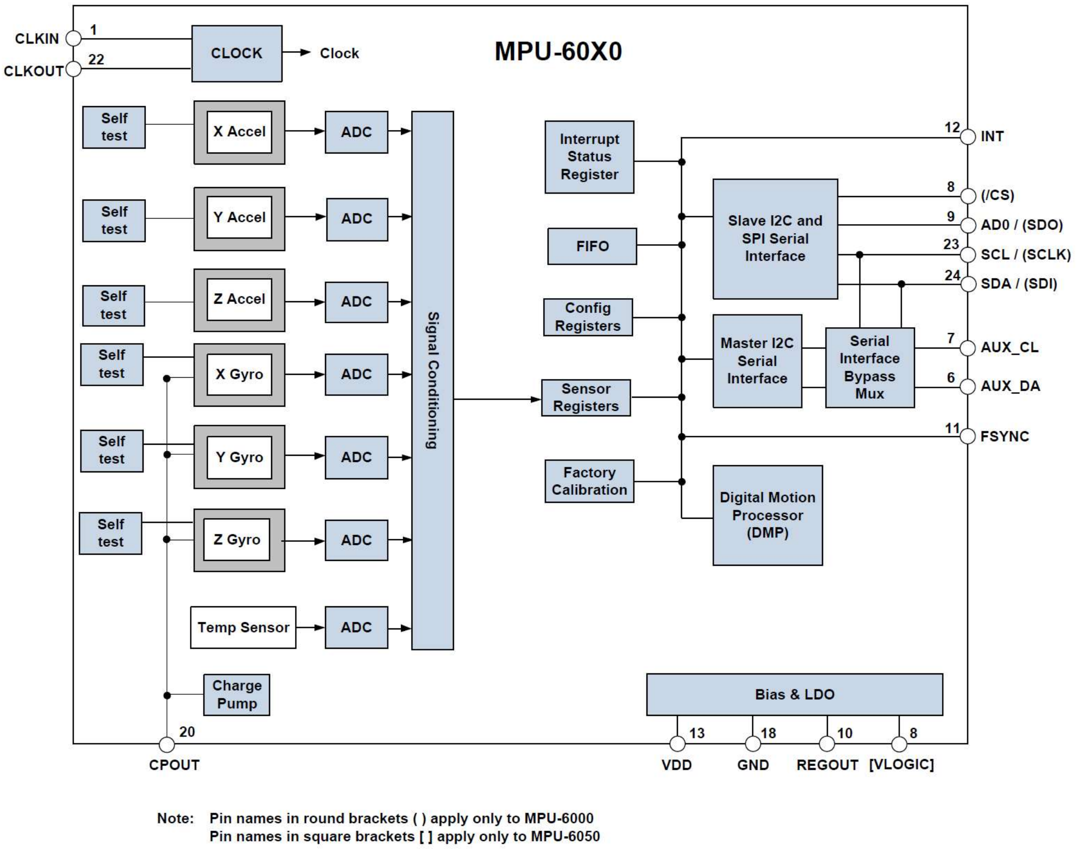

Figure S3. Block diagram of the inertial unit, MPU 6050 (InvenSense Inc.). .

Table S1. Pseudocode process of the 2D CNN-based feature extractor.

**Algorithm: The training process of the 2D CNN-based feature extractor**
**Input**: 6-dimension sequence data *X* (each head posture).
Output: Predicted head posture *Y*.
1. Sample *X* from head posture dataset;
2. Feed (*X, Y*) to the proposed model;
3. Update the feature extractor with the loss functions L_{tr}
4. Update the feature classificator with the loss functions L_{ce};
5. Train the 2D CNN-based feature extractor until convergence, thus obtaining the trained head posture *Y*.

Table S2. Comparison of previous methods for human activity recognition with our method.

Method	Sample type	Sampling method	Recognition ability	Wearable collection device	Wireless measurement of data
GMMs& accelerometer &EMG [21]	1D data	Direction measurement	√	√	×
Deep learning &camera [25]	Image	Image estimation	√	×	×
Descriptor fusion & camera [16]	Image	Image estimation	√	×	×
Gyroscope [38]	1D data	Direction measurement	×	√	×
POSI &camera [39]	Image	Angle estimation	√	×	×
Ion swarm &camera [40]	Image	Image estimation	√	×	×
DRRF &camera [41]	Image	Image estimation	√	×	×
ICP &camera [42]	Image	Image estimation	√	×	×

Information Sheet and Consent Form Checklist

This checklist is meant for helping the researchers to design their consent form. Researchers are suggested to provide the detailed information for the following items/areas. Please modify and edit the consent form in accordance with the requirements of the discipline concerned.

Format of the Informed Consent Form:
Consent in writing should be obtained. The informed consent form can be in a standard checklist format or letter format, as appropriate.

<u>Items for inclusion in the Consent Forms</u>

Tests of an attabchable device on the neck skin

Information of the Principal Investigator / Researcher

Name Hongcheng Xu
Department/Unit School of Mechano-Electronic Engineering
Institution: Xidian University
Contact Information (Address, Telephone, Fax, Email) xuhongcheng@stu.xidian.edu.cn

Invitation to participate
Please give a brief statement to invite the participant to take part in a research study. Please state clearly the collaborating parties and/or sponsor (if any).

A wearable device will be patched on a participant's back neck for recording the corresponding byphysical information. The sensor encapsulated by the silicone layer is nontoxic and harmless to human body.

Description of the study
The information sheet should set out the purposes/aim of the study, procedures and participant's time involvement / commitment in layman's language.

- Purpose: Please explain the purposes/aims of the study.

  To investigate the sensing properties of a wearable device, the sensor was conformal with the empidermis skin of a particitant's back neck to record the corresponding biophysical features. These tests is only to show the applied ability in physical situation。

- Procedures: Please describe procedures in details and explain what a participant will experience and be asked to do, provide a description of the devices and techniques to be used, if applicable.

  The wearable devices sensor will be patched with the skin of a particitant's back neck, and the sensor is wireless connected with a APP to record the

inertial motions, hence refecting head movements. In the tested process, the participant will not do anything, just turn over theis head as predetermined postures.

- Please state the tool or test to be used for evaluating the participant's physical or mental or cognitive ability, in particular for the vulnerable subjects, if applicable.

  In the tested process, the medical adhesive tape maybe form some little uncomfortable because of the adhesive tape on skin. However, this influence will elimate soon after the tape is took off, and this process is nontoxic and harmless to human body.

- Please mention if photography, video/audio recording will be used during the study. Please explain who will have access, what will become of tapes/files after use, e.g. shown at scientific meetings, describe when and how the tapes/files will be erased or destroyed, if applicable.

  In the tested process, photography will be used during the study. These photos will be public in the scientific published paper in future. As these photos will not be public some key privacies, like appearance.

Risks and Benefits

(i) Potential risks/discomforts and their minimization:

- Please describe the associated, potential or foreseeable risks or discomforts that a participant may experience during the study.
- Please describe how the risks/discomforts can be minimized. Where and how the participants can seek help to deal with any negative consequences / effects arising from the procedures.
- If there is no risk, please state as such.

There is no risk. The sensor was just patched on the neck's skin, and the sensor is nontoxic and harmless. The medical adhesive tape maybe form some little uncomfortable because of the adhesive tape on skin, so this test will no risk.

(ii) Potential benefits: please describe any benefits to the participant and others, if applicable.

The participant's name will be added to the author list.

Compensation for participation / Payments (if any):

Please describe the compensation/payment that the participant will receive for his or her participation in the study, if any.

In this study, the participant is the member of our group, so there is no payments.

Participant's Right - Participation and withdrawal

Please inform the participant that his/her participation is entirely voluntary and that he/she has the right to withdraw from the study any time without any negative consequences, penalty, or loss of benefits to which he/she is otherwise entitled.

Privacy and Confidentiality

Please assure the participant that any information that is obtained in connection with the study will be used for research purpose only. Unless the participant's prior permission is obtained or it is required by law, the identifiable information or identity of the participant will remain confidential and will not be disclosed. Detailed information should be provided to the participant when seeking such permission.

Please describe how confidentiality will be maintained, e.g. coding procedures, plans to safeguard data, including where data will be kept, who will have access …etc.

There is no confidentiality.

If the data collection/research work will be outsourced or subcontracted to the outsider, please state clearly the name of the organization/service provider involved, how confidentiality will be maintained by the service provider and the research team. Please state the details of the service provider's data handling policy; if not, please assure the compliance with the CityU's ethical standard by the service provider. Besides, please state clearly the ownership of the data collected, data retention plan, data access request etc.

These collected data and photos own to the Hongcheng Xu's group.

If information obtained will be released to any other party for any reason, please state clearly the purpose of the disclosure, the person/agency/organization to whom the information will be released, and the conditions under which it will be released.

In the future, there data and photos will be published in one scientific jounal and these information will be then released to the corresponding journal.

Please explain if the result will be reported as group results. If the study group result can be made available to participants/parents/third parties, please include a statement informing the interested parties how to obtain a copy of the result.

Yes, these results will be reported as group results. The participant will be available to download the corresponding paper once published in a journal.

Version A
(for use by Adult Participant)

Reply Slip

I have read the above information statement and understand the procedures and other information described above.
I will participate in the research entitled "Attachable Inertial Device with Machine Learning toward Head-Posture Monitoring in Attention Assessment".
(** Please delete if inappropriate)

OPTIONAL
Items included in this table are optional. Please delete if inappropriate.

I give consent:

• to be audiotaped during this study*	____yes__√___no
• to be videotaped during this study*	____yes__√___no
• to be photographed during this study*	__√__yes_____no
• for audio/video tapes/files, photos resulting from this study to be used for (describe the proposed use of tapes/files/materials)*.	___yes__√____no
• for recording the online activities during this study.	____yes__√___no
• for my identity to be revealed in written materials (and or other medium, please specify) resulting from this study.	____yes___√__no

Ying Peng Chao He

____
Signature of Participant

Hongcheng Xu.
____
Printed Name of Participant

Date 6th Dec 2022
